# Age Structure and Body Size of the Plateau Brown Frog (*Rana kukunoris*) in the Jiuzhaigou National Nature Reserve and Potential Climatic Impacts on Its Life History Variations

**DOI:** 10.3390/ani13233654

**Published:** 2023-11-25

**Authors:** Meihua Zhang, Cheng Li, Peng Yan, Bingjun Dong, Jianping Jiang

**Affiliations:** 1 China-Croatia “Belt and Road” Joint Laboratory on Biodiversity and Ecosystem Services, Chengdu Institute of Biology, Chinese Academy of Sciences, Chengdu 610041, China; zhangmh@cib.ac.cn (M.Z.); licheng@cib.ac.cn (C.L.); ypzz8899@163.com (P.Y.); 2 College of Life Sciences, Shenyang Normal University, Shenyang 110034, China

**Keywords:** Jiuzhaigou National Nature Reserve, *Rana kukunoris*, age structure, body size, skeletochronology, climatic impacts

## Abstract

**Simple Summary:**

Knowledge of life history traits is crucial for understanding population dynamics, biodiversity declines, and conservation management decisions. Here, we quantified the age structure and body size of the plateau brown frog (*Rana kukunoris*) in the Jiuzhaigou National Nature Reserve (JNNR), providing the first data about the life history traits of this species in this region. Subsequently, we compared the maximum longevity, age at sexual maturity (ASM), average age, and average snout–vent length (SVL) with those of 28 reported populations, and we examined the climatic influences on these four key life history traits. Notably, the maximum longevity in the JNNR population is 8 years, reaching the reported maximum longevity of this species. As elevation increases, the average age and ASM also increase. However, the average SVL initially increases before decreasing when above an elevation of 3000 m, which does not support Bergmann’s rule. Climatic factors, particularly temperature and UV-B, have discriminative effects on the life history variations of *R. kukunoris*. Our results will contribute to a deeper understanding of the diverse life history strategies and the related driving forces within a species.

**Abstract:**

Jiuzhaigou National Nature Reserve (JNNR) is a renowned World Biosphere Reserve and UNESCO-designated World Nature Heritage Site. The age structure and body size of a population are crucial for assessing the quality of habitats in which a population lives and are essential for the vertebrate conservation and management, especially for amphibians. Unfortunately, information about the life history traits of amphibians is currently unavailable in JNNR. Herein, we first estimated the age structure and body size of *Rana kukunoris*, which is endemic to the Eastern Qinghai-Xizang Plateau. Then, we compared our data with 28 reported populations along an elevation gradient (1797–3450 m) and investigated how life history traits respond to climatic variations. Our results indicated the following: (1) For individuals from JNNR, the maximum longevity is 8 years, age at sexual maturity (ASM) is 2 years, suggesting a favorable ecological environment in JNNR. Notably, females are significantly larger than males due to the age factor. (2) The average age and ASM show a positive correlation with elevation. However, when the elevation exceeds 3000 m, the average SVL initially increases and then decreases due to the harsh environmental conditions at higher elevation. (3) Temperature and/or UV-B have a significant impact on the average age, ASM, and average SVL variations of *R. kukunoris*, suggesting adaptive potential of this species via life history variations in light of environmental changes. These accounts provide antecedent information about the life history traits of amphibians in JNNR, and provide insights into the driving factors of the life history variations of the plateau brown frog.

## 1. Introduction

Understanding amphibian life history traits and their response to the changing climate is a priority for the identification of targeted conservation activities [1]. Amphibians are the most threatened vertebrate class, with 40.7% of species being globally threatened [2]. Their biodiversity is declining more rapidly than birds and mammals due to physiological constraints, aquatic and terrestrial life history, and limited dispersal capacity [3]. Climate change has been an ongoing and projected threat to amphibian biodiversity, as are habitat loss, disease, overutilization, and pollution [4,5,6]. It is well established that knowledge of life history traits is essential for assessing population dynamics and biodiversity declines, and for developing effective conservation strategies for amphibians [7,8]. Specifically, the life history of an organism encompasses its lifetime pattern of growth, development, reproduction, and survival patterns [9]. Age structure and body size are vital components for studying a population’s life history [10]. Age structure directly reflects upon growth rate, age at sexual maturity, and longevity, which are intimately linked to fitness attributes such as survival and reproductive output [11]. Moreover, age structure provide essential insights for assessing population status, forecasting future trends [12]. The population size and fluctuations are important reference indicators for determining the conservation status of taxa and the need for protected areas [13,14]. Body size is a highly variable trait that is affected by age, gender, phylogeny, and the environment; conversely, it influences various life history characteristics and numerous ecological and evolutionary processes such as geographic range, dispersal ability, and reproductive strategies [15,16,17].

The Jiuzhaigou National Nature Reserve (JNNR, 32.900–33.266° N, 103.767–104.050° E, 1996–4764 m above sea level) is a World Biosphere Reserve and a UNESCO (United Nations Educational, Scientific and Cultural Organization)-designated World Nature Heritage Site. It lies between the eastern rim of the Qinghai–Xizang Plateau and the Sichuan Basin, covering an area of 651 km^2^ [18]. This reserve is dedicated to the conservation of nationally protected animals such as the giant panda, golden monkey, and takin, as well as their habitats and unique water ecosystems [19]. According to Qiao et al. (2016), the annual air temperature in the Jiuzhaigou region has increased by 1.2 °C from 1951 to 2014, contributing to the current degradation of the tufa landscape [20]. However, the impact of climate change on vertebrates in JNNR remains unclear. In fact, amphibians are excellent biological indicators for exploring the effects of climatic change on vertebrates, as they are highly sensitive to subtle changes in surrounding environments [21]. The amphibian biodiversity in JNNR is relatively low due to the challenging alpine environment, which is characterized by low temperatures, strong temperature fluctuations, oxygen deficits, and high ultraviolet radiation [18]. To date, only five amphibian species have been recorded there: *Batrachuperus tibetanus* Schmidt, 1925 [22] (Urodela, Hynobiidae Cope, 1859 [23]), *Scutiger boulengeri* (Bedriaga, 1898) [24] (Anura, Megophryidae Bonaparte, 1850 [25]), *Bufo gargarizans* Cantor, 1842 [26] (Anura, Bufonidae Gray, 1825 [27]), *Rana kukunoris* Nikolskii, 1918 [28] (Anura, Ranidae Batsch, 1796 [29]), and *Amolops mantzorum* (David, 1872 [30]) (Anura, Ranidae Batsch, 1796 [29]). Unfortunately, information about their life history traits is currently unavailable.

Variations in life history traits among the populations of the same species could provide an opportunity for assessing the adaptive potential of amphibians to climatic variables, particularly for widespread species [31,32]. The plateau brown frog (*R. kukunoris*) is endemic to the Qinghai–Xizang Plateau of Southwest China at elevations ranging from 2000 to 4400 m [33]. This species occupies plateau grasslands, marshes, and seasonal ponds, playing a crucial role in the structure and function of wetland ecosystems [34]. As a typical explosive breeder, it breeds from March to May, with a breeding duration lasting for only 9–21 days [35]. The researches on the plateau brown frog have focused on activity characteristics [36,37,38], phylogenetic relationships [39,40,41], life history traits [35,42,43,44], and morphological and molecular adaptations to the harsh plateau environment [45,46,47]. The plateau frog has been selected as an ideal model for understanding potential responses to climatic change, and research studies have been conducted on the age structure and body size variations of 28 populations of this species [42,43,44]. However, Chen et al. (2011) note that, apart from the duration of the annual period of activity, other environmental and genetic factors influencing age and body size need to be addressed in further studies [42].

In the present study, we aim to (1) quantify the age structure and body size of *R. kukunoris* in JNNR to provide the first accounts about the life history traits of this species, (2) compare these results with the data from 28 previously reported populations distributed along an elevation gradient (~1790–3450 m) to assess the environmental quality of JNNR, and (3) investigate how its life history traits respond to the climatic factors across geographic ranges.

## 2. Materials and Methods

### 2.1. Sampling

**Figure 1:**
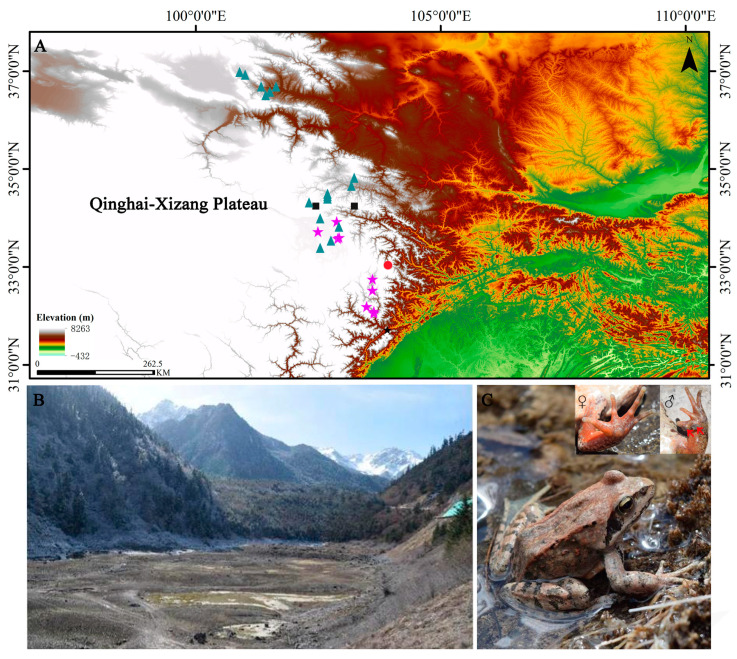
The studying site of *Rana kukunoris*, newly sampled in this work, is marked by the red circle (**A**). Other markers are for comparison. Specifically, the other sites were obtained from published literature, including Chen et al. (2011) [42], Feng et al. (2015) [43], and Yu et al. (2021) [44], and are marked by black squares, violet stars, and dark green triangles, respectively (**A**). Supplementary Materials Table S1 listed the detailed site information including the latitude, longitude, and elevation. The habitat (**B**) and an adult female photograph (**C**) of *R. kukunoris* in the Jiuzhaigou National Nature Reserve. The two red arrows indicate the nuptial pads.

This study was conducted at the upper seasonal lake (33°3′16″ N, 103°55′43″ E, 2909 m a.s.l.) in the Jiuzhaigou National Nature Reserve, Sichuan Province, China (Figure 1). This study area is free from human interference, as people rarely visit the area. In total, 101 individuals were randomly collected during the breeding season on 13–24 April 2021. First, sex was determined based on external morphological characteristics. Adult males have a larger body size with mostly pink or yellow-white abdomen, and more importantly, they possess well-developed gray nuptial pads at the base of each finger II (Figure 1C, ♂). Adult females also have a larger body size with generally reddish brown or orange-red abdomen, and lack the nuptial pads (Figure 1C, ♀). The juveniles have a relatively smaller body size and lack external secondary sexual characteristics [34]. Secondly, the snout–vent length (SVL) and body mass (BM) of each individual were measured utilizing a digital caliper, with results rounded to the nearest 0.01 mm, and an electronic scale, with results rounded to the nearest 0.01 g. Thirdly, the terminal phalanx of the longest right toe, toe IV, of each individual was clipped and preserved in a 4% paraformaldehyde solution for skeletochronology. Before being released at the point of capture, the iodine solution (0.5%) was used to disinfect the amputated toe to prevent inflammation.

### 2.2. Skeletochronology

Skeletochronology is an excellent tool for evaluating age structure without sacrificing specimens. Skeletal tissue sections were prepared as reported [48]. Briefly, (1) bone decalcification was carried out: The outer skin and muscle tissue of each phalange were removed, soaked in running water for 2 hours, decalcified in 5% nitric acid for 48 h, and rinsed under running water for 12 h. (2) Staining and dehydration were followed: The phalanges were stained in Ehrlich’s hematoxylin for 75 min, and then dehydrated in 75%, 80%, 90%, and 100% alcohol concentration for 1 h at each concentration. (3) Paraffin embedding and sectioning were carried out: Tissues were embedded in paraffin blocks and sectioned at 13 μm with a rotary microtome. All sections were observed, accounted for growth marks, and photographed at 40× magnification under the optical microscope (Optec B302, Chongqing Optec Instrument Co., Ltd., Chongqing City, China) equipped with a CCD camera (ICX285A, Sony, Tokyo City, Japan).

The surface of the bone was counted as a valid LAG, because all specimens were collected after hibernation (LAG usually develops when anuran hibernates). False lines are usually fainter than the LAGs and cannot form a complete closed loop in the cross-section of the bone, and double lines are recorded as one LAG. Endosseous resorption usually affects the age line count. Thus, we used the back-calculation method (BCM) to detect whether an individual has experienced endosteal resorption [49]. The specific method was used to calculate the mean value of the diameter of the first LAG of all samples, and then the diameters of the first LAGs of other samples were compared. If the diameter difference was greater than 2SD, endosteal resorption has occurred [50]. To ensure the credibility of the counting results, three researchers independently counted the LAGs without knowing the SVL and BM data, and the counts were averaged to obtain the mean variable.

### 2.3. Climatic Variables

The temperature, precipitation, and ultraviolet-B (UV-B) radiation are often considered the most critical factors affecting the life-history traits, particularly for amphibians [51,52]. We used an initial set of 23 climatic predictors, including 19 bioclimatic variables (bio1~19) and 4 UV-B variables (UV-B1~4) as environmental predictors to explore the climatic impacts on life history variations among 29 populations of *R. kukunoris* (Table S1). Bioclimatic and UV-B data were obtained from the WorldClim [51] and gIUV datasets [52] by utilizing ArcGIS 10.7 (ESRI, Redlands, CA, USA), respectively. To avoid multicollinearity of these climatic predictors [53], we examined cross-correlation of the 23 variables and eliminated the highly correlated (|Pearson *r*| ≥ 0.8) climatic variables (Figure S1) [54]. Finally, we retained five environmental variables, including the annual mean temperature, mean monthly temperature range, isothermality, annual precipitation, and annual mean UV-B (Table S2), which explained 80.57% of the total variance based on principal component analysis (PCA) with an eigenvalue threshold of >1.0 (Table S3).

### 2.4. Statistical Analyses

The differences in age structure and body size between both sexes of *R. kukunoris* were analyzed via the Mann–Whitney U test. Next, a linear regression model was utilized to estimate the relationship between age and body size in adult males and females, and to examine the environmental effects on life history variations. To identify the importance, effect, and independent contribution of each selected environmental factor to the life history variations among 29 localities, we conducted hierarchical partitioning using the hier.part package [55,56]. Multiple regression was conducted to determine the combined impact of the five environmental factors on life history variations, and significance was tested with ANOVA analysis. All statistical analyses were performed in R 4.2.3 [57]. The values were presented as mean ± SD. All probabilities were two-tailed, and the level of significance was *p* < 0.05.

## 3. Results

### 3.1. Age Structure and Body Size of R. kukunoris in JNNR

**Figure 2:**
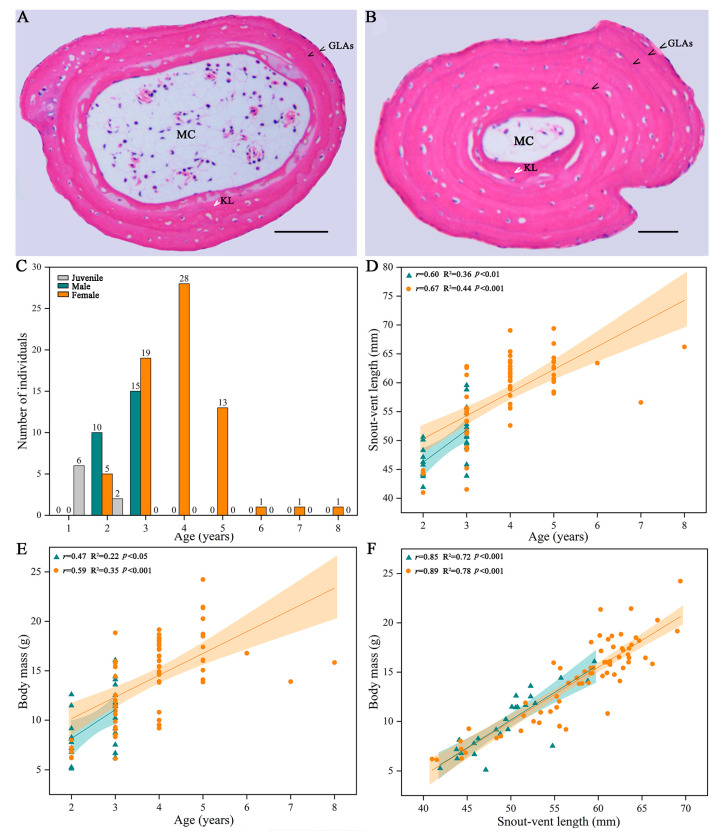
Representative phalangeal growth marks in cross sections of an adult male *R. kukunoris* at an age of 2 years (**A**), an adult female at an age of 4 years (**B**), age distribution (**C**), relationship between age and snout–vent length (**D**) and body mass (**E**), relationship between snout–vent length and body mass (**F**). Black arrows indicate lines of arrested growth (LAGs), and white arrows indicate the Kastschenko Line (KL). The scale bar is 100 μm. Abbreviations: KL = Kastschenko Line, LAG = line of arrested growth, mc = medullar cavity.

We obtained the ages of 101 individuals via skeletochronology, including 8 juveniles, 25 males, and 68 females (Table 1). Both sexes reached sexual maturity during the second year after metamorphosis (2 years). The adult male age ranged from 2 to 3 years, with a majority of 3 years (60%), while the adult female age ranged from 2 to 8 years, with a majority of 3 (27.94%), 4 (41.18%), and 5 years (19.12%) (Figure 2A–C). The average age of females was significantly older (3.90 ± 1.09 years) than males (2.60 ± 0.50 years) (Table 1).

Results of linear regression analyses showed that, age was significantly and positively correlated with SVL (Figure 2D) and BM (Figure 2E), the SVL was strongly and positively correlated with the BM (Figure 2F), and the growth rates of the males (SVL: y = 5.55 age + 35.13, R^2^ = 0.36, *p* < 0.01; BM: y = 2.87 age + 2.45, R^2^ = 0.22, *p* < 0.05) were higher than that of the females (SVL: y = 3.99 age + 42.31, R^2^ = 0.44, *p* < 0.001; BM: y = 2.20 age + 5.74; R^2^ = 0.35; *p* < 0.001).

### 3.2. Comparisons of the Average Age, ASM, and Average SVL among 29 Populations of R. kukunoris

**Figure 3:**
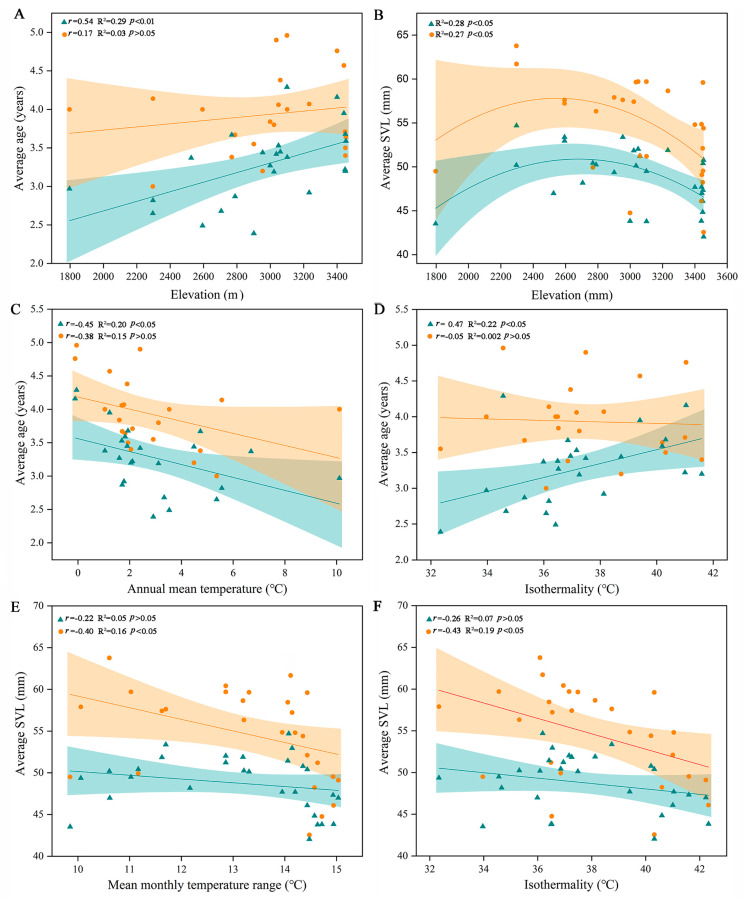
Variation in average age (**A**) and average SVL (**B**) of *R. kukunoris* along an elevational gradient (~1790–3450 m) on the Qinghai–Xizang Plateau, annual mean temperature (**C**) and isothermality (**D**) impact on the average age, the mean monthly temperature range (**E**) and isothermality (**F**) impact on the average SVL. Green indicates males, and red indicates females.

The maximum longevity of *R. kukunoris* was 7 years for males and 8 years for females. The ASM was 2–4 years old (Table S1). The average male age significantly increased relative to elevation, while this did not occur for females (*p* > 0.05; Table 2; Figure 3A). The ASM of both males and females was significantly correlated with elevation (*p* < 0.01; Table 2). However, the average SVL of both sexes was not significantly and linearly correlated with elevation (*p* > 0.05; Table 2). Specifically, with an increase in elevation, the average SVL increased first and then decreased significantly (*p* < 0.05), presenting a hump shape (Figure 3B).

The linear regression models showed that the annual mean temperature (*r* = −0.45, R^2^ = 0.20, *p* < 0.05) and isothermality (*r* = 0.47, R^2^ = 0.22, *p* < 0.05) had a significant impact on the average age of the males, while these environmental predictors had little impact on the average age of the females (*p* > 0.05; Figure 3C,D). All selected environmental predictors had significant impacts on the age at sexual maturity with respect to both sexes, except for annual precipitation (*p* > 0.05; Table 2). The average SVL of the females was negatively influenced by the mean monthly temperature range (*r* = −0.40, R^2^ = 0.16, *p* < 0.05) and isothermality (*r* = −0.43, R^2^ = 0.19, *p* < 0.05), while that of the males was not significantly influenced by these environmental predictors (*p* > 0.05; Figure 3E,F).

### 3.3. The Environmental Impacts on the Life History Variations of These Populations

The mean monthly temperature range, isothermality, and annual mean UV-B were significantly and positively correlated with the elevation gradient (*p* < 0.001), while the annual mean temperature was significantly and negatively correlated with the elevation gradient (*p* < 0.001; Table S4).

**Figure 4:**
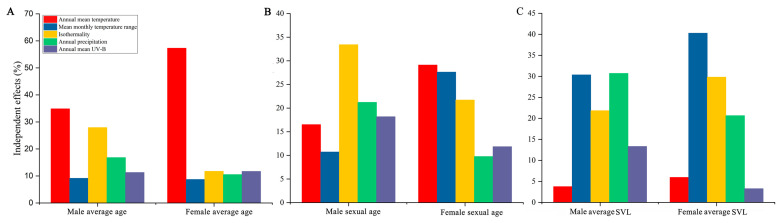
Independent contribution of climatic effects on average age (**A**), age at sexual maturity (**B**), and average snout–vent length (**C**) of male and female *R. kukunoris*.

Multiple regression models showed that mixed environmental predictors had significant impacts on the ASM and average SVL in both sexes (*p* < 0.05; Table 3). Hierarchical partitioning analyses revealed that the annual mean temperature contributed the most to the average age in males (34.87%) and females (57.32%); for the ASM, isothermality contributed the most in males (33.40%), while the annual mean temperature contributed the most in females (29.11%); for the average SVL, annual precipitation contributed the most in males (30.71%), while the mean monthly temperature range contributed the most in females (40.28%) (Table 3; Figure 4).

## 4. Discussion

Studies have demonstrated that the age structure and body size of a population are crucial for assessing the quality of habitats where the population lives, and are essential for their conservation and management, especially for amphibians [58,59]. The maximum longevity of *Rana temporaria* Linnaeus, 1758 is 18 years, which is the longest lifespan ever reported for a common wild frog of the family Ranidae [60]. In the JNNR population, *R. kukunoris* has a lifespan of 8 years, which is consistent with the maximum longevity recorded for populations of this species at an elevation of 3100, 3400 [42], and 3441 m [44]. This life history trait indicates that frogs in JNNR have sufficient food, few predators, and a favorable ecological environment, providing direct insights into the importance of protected areas in offering refuge for herpetofauna from climate change [61]. During reproduction seasons, *R. kukunoris* males prefer to select larger females for mating [62]. Fecundity selection suggests that females of a larger size possess greater abdominal cavity for accommodating more offspring and energy storage, thereby increasing reproductive output [63]. Sexual size dimorphism is influenced by several factors such as size at metamorphosis, growth rate, and age [64,65]. In the JNNR population, both males and females reach sexual maturity at an age of 2 years, and there are no significant sexual differences in body size even at an age of 3 years. Additionally, the growth rate of males is higher than that of females, which is in accordance with the findings of previous studies on this species [43,44]. Therefore, our study suggests that female-biased sexual size dimorphism in *R. kukunoris* is driven by age. The maximum age of males in the JNNR population is 3 years, which is lower than that in other populations [42,43,44]. To our knowledge, *R. kukunoris* males have a nearly 1.7 times greater activity range than females around the breeding sites, making it harder to collect enough male specimens [66]. Moreover, after reproduction, larger males migrate to spring and grassland habitats that are far from breeding ponds to obtain food, benefiting from their relatively smaller surface area and lower water loss rate. In contrast, smaller individuals are confined to foraging around ponds due to their relatively larger surface area and higher water loss rate, particularly for seasonal breeding sites [37].

Notably, the life history plasticity of *R. kukunoris* varies in response to the changing climatic variables. For 29 *R. kukunoris* populations, the average age and age at sexual maturity of both sexes increased as elevation increased. Our study reveals that as elevation increases, the annual mean temperature significantly decreases, while the mean monthly temperature range, isothermality, and annual mean UV-B significantly increase. The harsh climate conditions of high elevations result in fewer competitors, reduced competition intensities, shorter active seasons, and longer hibernation periods, which help animals avoid predation risks and food shortages. Additionally, rate living theory indicates that, for ectotherms, colder temperature results in reduced metabolism and lower intrinsic mortality due to metabolic by-products (e.g., spontaneous chemical reactions, replication errors, and oxidative damage) [67,68]. These extrinsic and intrinsic factors contribute to the larger average age of the plateau frog [69,70]. The life history strategy is determined via the trade-offs between traits related to growth, reproduction, and survival [9]. Individuals at higher elevations mature later, which is a prerequisite for self-maintenance and survival in the harsh plateau environment [71]. Interestingly, the average SVL of *R. kukunoris* in each sex increases initially and then decreases with an increase in elevation, and this pattern also occurs in *R. temporaria* with an increase in latitude [72]. This is in contrast with Bergmann’s rule, which predicts that organisms living in colder environments should have larger body sizes [73]. The reduced body size may be attributable to the shorter growing season at the higher site. Indeed, the plateau’s extreme environmental factors pose huge challenges to individual survival at elevations above 3000 m, including shorter activity periods, food shortages, and intense interspecific competitions [10]. Additionally, growth is generally more costly for ectotherms living in environments where activity time is a limited resource [10].

However, climatic factors have varying effects on the life history traits of *R. kukunoris*. Annual precipitation has a minimal impact on the average age, ASM, and average SVL of *R. kukunoris*. Similarly, the tadpole survival rate and relative metamorphosis rate of *R. kukunoris* are not correlated with rainfall amounts [74]. This may be because this species lives in close proximity to permanent aquatic environments, resulting in a minimal impact of precipitation. It has been reported that there is a significant negative correlation between individuals encountered and the distance to the aquatic site [37]. In autumn, these frogs stop moving around seasonal breeding ponds, and instead begin migrating toward constant flowing water in order to overwinter [38]. The annual mean UV-B exhibits a significant positive correlation with the age at sexual maturity in both sexes. This finding supports the result that relatively high UV-B radiation can promote the amphibian growth, especially for amphibians at earlier life history stages [75]. In recent years, climate change has collectively led to an increase in the frequency, intensity, and duration of extreme weather events worldwide [76]. Long-term monitoring of the life history traits of *R. kukunoris* will contribute to a better understanding of how amphibians respond to global climate change and to provide basic reference data for conservation activities [77].

## 5. Conclusions

This study provides first-hand insights into the life history traits of *R. kukunoris* in JNNR. The longevity of the JNNR population is 8 years, reaching the reported maximum longevity of this species and indicating that there are favorable environmental conditions in JNNR. Climatic factors, particularly temperature and UV-B, play significant roles in driving the life history variations (i.e., average age, ASM, and average SVL) of *R. kukunoris*. These results contribute to enhancing our understanding of elevation-related variation in the life history features of plateau ectotherms and their life history plasticity and adaptive potential, providing an important basis for conservation management. Although annual precipitation exerts minimal impacts on life history traits, the warming climate and altered rainfall patterns induced by global climate change may ultimately influence the lifespan and other life history traits of the plateau brown frog in the future. To gain a better understanding of how amphibians respond to global climate change, we recommend continuous monitoring of the life history traits of *R. kukunoris*.

## Figures and Tables

**Table 1:** Age structure and body size of *R. kukunoris* in Jiuzhaigou National Nature Reserve. n indicates the studied individuals.

Age	Male			Female			Z	P	Z	P
	n	SVL (mm) (Mean ± SD)	BM (g) (Mean ± SD)	n	SVL (mm) (Mean ± SD)	BM (g) (Mean ± SD)	SVL		BM	
2	10	46.2 ± 2.8 (41.9–50.6)	8.2 ± 2.4 (5.1–12.6)	5	43.8 ± 1.6 (41.0–44.8)	6.9 ± 0.8 (6.2–8.0)	−1.35	0.18	−1.10	0.27
3	15	51.8 ± 4.3 (43.9–59.6)	11.0 ± 2.9 (6.3–16.1)	19	53.7 ± 5.5 (41.5–62.9)	12.0 ± 3.3 (6.1–18.8)	−1.23	0.22	−0.64	0.52
4	0	/	/	28	60.7 ± 3.4 (52.6–69.1)	15.4 ± 2.7 (9.2–19.2)	/	/	/	/
5	0	/	/	13	62.5 ± 3.2 (58.2–69.4)	18.0 ± 3.1 (13.9–24.2)	/	/	/	/
6	0	/	/	1	63.4	16.8	/	/	/	/
7	0	/	/	1	56.6	13.9	/	/	/	/
8	0	/	/	1	66.2	15.8	/	/	/	/
Total	25	49.6 ± 4.6 (41.9–59.6)	9.9 ± 3.0 (5.1–16.1)	68	57.9 ± 6.6 (41.0–69.4)	14.3 ± 4.1 (6.1–24.2)	−5.10	0.00	−4.49	0.00

**Table 2:** Relationships between environmental variables and age as well as body size of *R. kukunoris* based on Pearson correlation analysis. n indicates the number of populations.

Environmental Variables	Average Age		ASM		Average SVL	
	Male (n = 24)	Female (n = 22)	Male (n = 24)	Female (n = 21)	Male (n = 29)	Female (n = 27)
Elevation	0.54 **	0.17	0.68 ***	0.60 **	−0.25	−0.34
Annual mean temperature	−0.45 *	−0.38	−0.48 *	−0.68 **	0.07	0.06
Mean monthly temperature range	0.20	0.16	0.30	0.72 ***	−0.22	−0.40 *
Isothermality	0.47 *	−0.05	0.65 **	0.61 **	−0.26	−0.43 *
Annual precipitation	0.23	−0.21	0.32	−0.37	−0.20	−0.11
Annual mean UV-B	0.35	0.20	0.54 **	0.52 *	0.09	−0.17

Notes: *, **, *** mean *p* < 0.05, *p* < 0.01, and *p* < 0.001, respectively.

**Table 3:** Independent contribution for environmental effects (in percentage) on age structure and body size of 29 *R. kukunoris* populations, based on multiple regression and hierarchical partitioning analyses.

Sex	Life History Traits	Full Model (*r*^2^)	Annual Mean Temperature	Mean Monthly Temperature Range	Isothermality	Annual Precipitation	Annual Mean UV-B
Male	Average age	0.44	34.87	9.15	27.91	16.80	11.28
	ASM	0.64 **	16.51	10.71	33.40	21.21	18.17
	SVL	0.57 **	3.77	30.36	21.84	30.71	13.32
	Average age	0.21	57.32	8.72	11.73	10.54	11.68
Female	ASM	0.69 **	29.11	27.61	21.71	9.75	11.83
	SVL	0.45 *	5.97	40.28	29.82	20.66	3.27

Notes: *, ** mean *p* < 0.05 and *p* < 0.01, respectively.

## Data Availability

The raw data used in this study can be available on request from the corresponseing authors.

## Supplementary Materials

The following supporting information can be downloaded at: https://www.mdpi.com/article/10.3390/ani13233654/s1, Table S1: Information about the locations and life history traits of JNNR and 28 reported *R. kukunoris* populations. Latitude and longitude units have been standardized into degrees (°). /means information absent; Table S2: Environmental variables compiled to depict environment gradients for *R. kukunoris*; Table S3: The first two principal components (eigenvalue > 1.0) and factor loadings of principal component analysis; Table S4: The Pearson correlation analysis of the relationships between the environmental variables and the elevation based on 29 populations of *R. kukunoris*; Figure S1: Pearson’s correlation analysis for climatic variables.
