# Supplementary material for: Age Structure and Body Size of the Plateau Brown Frog (Rana kukunoris) in the Jiuzhaigou National Nature Reserve and Potential Climatic Impacts on Its Life History Variations
Source: Animals (Basel). 2023 Nov 25;13(23):3654. doi: 10.3390/ani13233654 (PMC10705176; doi:10.3390/ani13233654)
Supplement: Supplementary file 1 [file animals-13-03654-s001.zip › animals-2692866-supplementary.pdf]

Age Structure and Body Size of the Plateau Brown Frog (*Rana kukunoris*) in the Jiuzhaigou National Nature Reserve and Potential Climatic Impacts on Its Life History Variations

Meihua Zhang[1], Cheng Li[1], Peng Yan[1,2], Bingjun Dong[2,*] and Jianping Jiang[1,*]

[1]China-Croatia "Belt and Road" Joint Laboratory on Biodiversity and Ecosystem Services, Chengdu Institute of Biology, Chinese Academy of Sciences, Chengdu 610041, China

[2]College of Life Sciences, Shenyang Normal University, Shenyang 110034, China

*Corresponding authors:

Bingjun Dong, email: dongbingjun@synu.edu.cn; Tel: +86-024-86593325

Jianping Jiang, email: jiangjp@cib.ac.cn; Tel: +86-028-82890789

Table S1. Information about the locations and life history traits of JNNR and 28 reported *R. kukunoris* populations. Latitude and longitude units have been standardized into degrees (°). / means information absent.

Latitude (°)	Longitude (°)	Elevation (m)	Male average age (years)	Female average age (years)	Male ASM (years)	Female ASM (years)	Male SVL (mm)	Female SVL (mm)	References
31.71	103.91	1797	2.97	4.00	2	2	43.53	49.50	[1, 2]
32.06	103.64	2296	2.65	3.00	2	2	50.20	63.77	[1, 2]
36.65	101.633333	2297	2.82	4.14	2	3	54.70	61.71	[3]
36.566667	101.5	2495	/	/	/	/	52.96	57.22	[3]
32.08	103.64	2526	3.37	/	2	/	46.99	/	[1, 2]
36.666667	101.333333	2594	2.49	4.00	2	3	51.44	58.45	[3]
34.633333	103.183333	2706	2.68	/	2	/	48.18	/	[3]
32.51	103.61	2769	3.67	3.38	2	2	50.44	49.93	[1, 2]
36.483333	101.433333	2789	2.87	3.67	2	3	50.25	56.33	[3]
33.054444	103.928611	2909	2.39	3.55	2	2	49.57	57.88	This sduty
32.18	103.49	2954	3.44	3.20	3	3	53.38	57.62	[1, 2]
36.9	101	2999	3.27	3.84	2	3	43.82	44.76	[3]
32.74	103.6	3022	3.19	3.80	3	3	51.85	57.42	[1, 2]
34.783333	103.233333	3036	3.42	4.90	3	3	50.13	59.65	[3]
34.483333	102.683333	3049	3.53	4.06	3	3	52.03	59.70	[3]
34.466667	102.683333	3060	3.45	4.38	3	3	51.23	60.42	[3]
34.25	103.233333	3100	4.29	4.96	3	3	49.50	59.70	[4]
36.95	100.9	3101	3.38	4.00	2	/	43.79	51.20	[3]
34.366667	102.683333	3233	2.92	4.07	2	3	51.90	58.65	[3]
34.25	102.45	3400	4.16	4.76	3	4	47.70	54.80	[4]
33.366667	102.55	3440	/	/	/	/	43.84	46.10	[3]

34.283333	102.316667	3441	3.95	4.57	3	3	47.71	54.84	[3]
33.966667	102.55	3443	/	/	/	/	47.00	49.10	[3]
33.816667	102.916667	3448	/	/	/	/	44.85	48.24	[3]
33.92	102.87	3448	3.22	3.71	3	3	46.10	52.11	[1, 2]
33.58	102.9	3450	3.68	3.50	3	3	50.40	59.60	[1, 2]
33.71	102.49	3450	3.20	3.40	3	3	47.34	49.54	[1, 2]
33.6	102.93	3453	3.59	3.64	3	3	50.78	54.40	[1, 2]
33.516667	102.766667	3454	/	/	/	/	42.05	42.56	[3]

References

1. Feng, X.Y.; Chen, W.; Hu, J.H.; Jiang, J.J. Variation and sexual dimorphism of body size in the plateau brown frog along an altitudinal gradient. *Asian Herpetol. Res.* **2015**, *6*, 291–297.
2. Leung, K.W.; Yang, S. N.; Wang, X.Y.; Tang, K.; Hu, J.H. Ecogeographical adaptation revisited: morphological variations in the plateau brown frog along an elevation gradient on the Qinghai-Tibetan Plateau. *Biology* **2021**, *10*, 1081.
3. Yu, T.L.; Jia, G.; Sun, H.Q.; Shi, W.H.; Li, X.L.; Wang H.B., Huang, M.R.; Ding, S.Y.; Chen, J.P., Zhang, M. Altitudinal body size variation in *Rana kukunoris*: the effects of age and growth rate on the plateau brown frog from the eastern Tibetan Plateau. *Ethol. Ecol. Evol.* **2022**, *34*, 120–132.
4. Chen, W.; Yu, T.L.; Lu, X. Age and body size of *Rana kukunoris*, a high-elevation frog native to the Tibetan plateau. *Herpetol. J.* **2011**, *21*, 149–151.

Table S2. Environmental variables compiled to depict environment gradients for *R. kukunoris*.

Abbreviation	Environmental variables	Included in the study
**bio01**	**Annual mean temperature**	**Yes**
**bio02**	**Mean monthly temperature range**	**Yes**
**bio03**	**Isothermality**	**Yes**
bio04	Temperature seasonality	No
bio05	Max temperature of the warmest month	No
bio06	Min temperature of the coldest month	No
bio07	Temperature annual range	No
bio08	Mean temperature of the wettest quarter	No
bio09	Mean temperature of the driest quarter	No
bio10	Mean temperature of the warmest quarter	No
bio11	Mean temperature of the coldest quarter	No
**bio12**	**Annual precipitation**	**Yes**
bio13	Precipitation of the wettest month	No
bio14	Precipitation of the driest month	No
bio15	Precipitation seasonality	No
bio16	Precipitation of the wettest quarter	No
bio17	Precipitation of the driest quarter	No
bio18	Precipitation of the warmest quarter	No
bio19	Precipitation of the coldest quarter	No
**UVB1**	**Annual mean UV-B**	**Yes**
UVB2	UV-B seasonality	No
UVB3	Mean UV-B of the highest month	No
UVB4	Mean UV-B of the lowest month	No

Table S3. The first two principal components (eigenvalue > 1.0) and factor loadings of principal component analysis.

Variables	PC1	PC2
Annual mean temperature	-0.843	0.015
Mean monthly temperature range	0.880	-0.226
Isothermality	0.740	0.472
Annual precipitation	-0.305	0.946
Annual mean UV-B	0.837	0.180
Eigenvalue	5.6538	2.4029
Cumulative variance (%)	56.538	80.567

Table S4. The Pearson correlation analysis of the relationships between the environmental variables and the elevation based on 29 populations of *R. kukunoris*.

Environmental variables	Elevation (m)
Annual mean temperature	-0.83***
Mean monthly temperature range	0.63***
Isothermality	0.76***
Annual precipitation	0.13
Annual mean UV-B	0.82***

Notes: ***means $p < 0.001$.

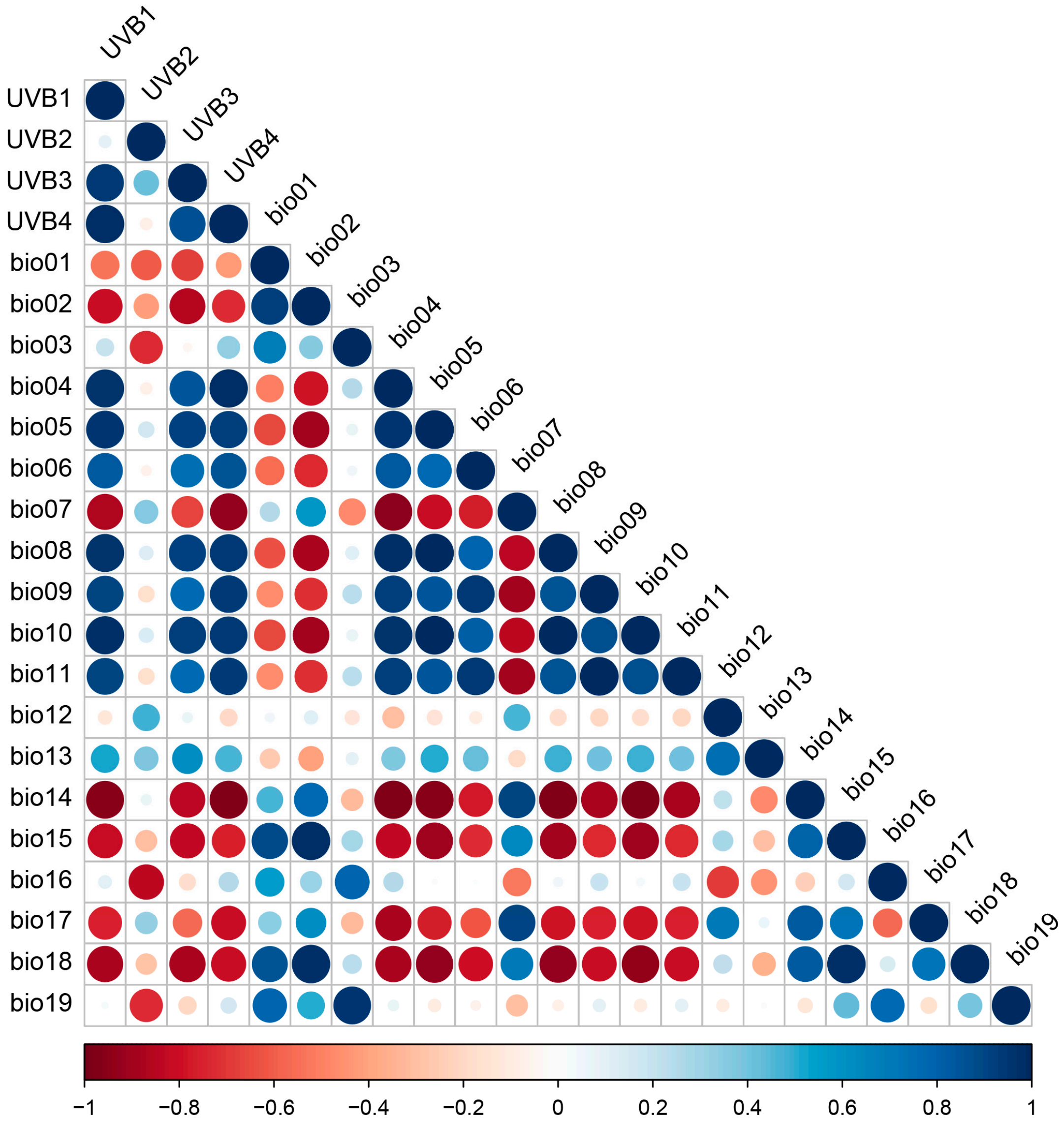

Figure S1. Pearson's correlation analysis for climatic variables.
